# Comprehensive Insights into Obesity and Type 2 Diabetes from Protein Network, Canonical Pathway, Phosphorylation and Antimicrobial Peptide Signatures of Human Serum

**DOI:** 10.3390/proteomes13040067

**Published:** 2025-12-17

**Authors:** Petra Magdolna Bertalan, Erdenetsetseg Nokhoijav, Ádám Pap, George C. Neagu, Miklós Káplár, Zsuzsanna Darula, Gergő Kalló, Laszlo Prokai, Éva Csősz

**Affiliations:** 1 Proteomics Core Facility, Department of Biochemistry and Molecular Biology, Faculty of Medicine, University of Debrecen, 4032 Debrecen, Hungarykallo.gergo@med.unideb.hu (G.K.); 2 Doctoral School of Molecular Cellular and Immune Biology, University of Debrecen, 4032 Debrecen, Hungary; 3 Laboratory of Proteomics Research, HUN-REN Biological Research Centre, 6726 Szeged, Hungary; 4 Single Cell Omics Advanced Core Facility, Hungarian Centre of Excellence for Molecular Medicine (HCEMM), 6728 Szeged, Hungary; 5 Department of Pharmacology and Neuroscience, College of Biomedical and Translational Sciences, The University of North Texas Health Science Center, Fort Worth, TX 76107, USAlaszlo.prokai@unthsc.edu (L.P.); 6 Department of Internal Medicine, Faculty of Medicine, University of Debrecen, 4032 Debrecen, Hungary

**Keywords:** obesity, type 2 diabetes, mass spectrometry, phosphoproteomics, network analysis, AMP

## Abstract

Background: Obesity is a major risk factor for type 2 diabetes (T2D); however, the molecular links between these conditions are not fully understood. Methods: We performed an integrative serum proteomics study on samples from 134 individuals (healthy controls, patients with obesity and/or T2D) using both data-independent (DIA) and data-dependent (DDA) liquid chromatography-mass spectrometry approaches, complemented by phosphopeptide enrichment, kinase activity prediction, network and pathway analyses to get more information on the different proteoforms involved in the pathophysiology of the diseases. Results: We identified 235 serum proteins, including 13 differentially abundant proteins (DAPs) between groups. Both obesity and T2D were characterized by activation of complement and coagulation cascades, as well as alterations in lipid metabolism. Ingenuity Pathway Analysis^®^ (IPA) revealed shared canonical pathways, while phosphorylation-based regulation differentiated the two conditions. Elevated hemopexin (HPX), vitronectin (VTN), kininogen-1 (KNG1) and pigment epithelium-derived factor (SERPINF1), along with decreased adiponectin (ADIPOQ) and apolipoprotein D (APOD), indicated a pro-inflammatory, pro-coagulant serum profile. Network analyses of antimicrobial and immunomodulatory peptides (AMPs) revealed strong overlaps between immune regulation and lipid metabolism. Phosphoproteomics and kinase prediction highlighted altered CK2 and AGC kinase activities in obesity, suggesting signaling-level modulation. Conclusions: Our comprehensive proteomic and phosphoproteomic profiling reveals overlapping yet distinct molecular signatures in obesity and T2D, emphasizing inflammation, complement activation and phosphorylation-driven signaling as central mechanisms that potentially contribute to disease progression and therapeutic targeting.

## 1. Introduction

Type 2 diabetes (T2D) is recognized as the most common metabolic disease affecting millions of people all around the world [1,2]. According to the International Diabetes Federation, the number of patients diagnosed with T2D will rise to 852 million by 2050 [2]. Many genetic and environmental factors play a role in the development of T2D [3]. Several genes, such as *PPARG*, *KCNJ1* and *TCF7L2* have been described, which primarily play a role in insulin secretion and its action [4]. Furthermore, the heritability of T2D has also been demonstrated [2,3,5]. However, the major risk factor for the development of T2D is obesity, which is arising with an increasing tendency as a consequence of unhealthy lifestyle choices, such as excessive dietary intake and a lack of physical activity, leading to an increase in body weight [1,2,6,7,8]. Obesity is mainly defined by the body mass index (BMI) where a BMI of ≥30 is considered obese [9]. Several studies have investigated the relationship between an increased BMI and the development of various diseases, including T2D [10,11], hypertension [9], cardiovascular disease (CVD) [12], kidney disease [13], osteoporotic fracture [14] and a number of cancers [15,16,17]. In addition to the strong link between elevated BMI and obesity-related diseases, high blood glucose levels and insulin resistance can lead to dyslipidemia and high triglyceride levels, as well as inflammation [18,19], all of which further contributes to cancer [20] and CVD [21].

Many studies apply proteomics for the examination of obesity and T2D. In metabolically abnormal obesity, results noted the reduced level of albumin (ALB), hemoglobin subunit alpha (HBA1), hemoglobin subunit beta (HBB), C-reactive protein (CRP), serum paraoxonase/arylesterase 1 (PON1) and haptoglobin-related protein (HPR), while an increased level of alpha-2-HS-glycoprotein (AHSG) was reported [22,23,24]. Multiple studies have also investigated the proteomic landscape of T2D risks. While insulin-like growth factor-binding protein complex acid labile subunit (IGFALS), insulin-like growth factor-binding protein 3 (IGFBP3) and insulin-like growth factor 2 (IGF2) revealed a negative correlation with elevated BMI [25], leptin (LEP), fatty acid binding protein 4 (FABP4) and insulin-like growth factor-binding protein 1 (IGFBP1) were positively correlated with increased risk of T2D development [26]. The elevated level of transthyretin (TTR) and the decrease of ALB have also been reported in T2D [27]. A study published in 2023 by Li et al. [28] has proposed potential biomarker candidates in serum for T2D: vitamin D-binding protein (GC), apolipoprotein B-100 (APOB), apolipoprotein A2 (APOA2), apolipoprotein A1 (APOA1), TTR, immunoglobulin heavy variable 3-13 (IGHC3-13), antithrombin-III (SERPINC1), fibrinogen gamma chain (FGG), fibrinogen alpha chain (FGA) and alpha-1-antitrypsin (SERPINA1). These studies have revealed the potential of proteomics in future T2D research. However, the identification of body fluid proteins can be challenging due to their heterogeneous composition and the wide dynamic range of protein concentrations [29].

In order to address some of the above-mentioned analytical challenges, proteomics rely mostly on mass spectrometry (MS) due to its exceptional selectivity, sensitivity and low detection limits [30,31]. Bottom-up shotgun proteomics aims at generating comprehensive data on the entire proteome through liquid chromatography–tandem mass spectrometry (LC–MS/MS) [32,33]. Data acquisition in shotgun proteomics can be performed using either data-dependent acquisition (DDA) or data-independent acquisition (DIA) methods [33,34]. The primary distinction between these two approaches lies in the selection of precursor ions for fragmentation. During DDA, we select a subset of precursor ions for fragmentation (usually the most abundant ones) while we fragment all precursor ions within predetermined m/z isolation windows during DIA [33,34].

MS analyses have identified a number of antimicrobial and immunomodulatory peptides (AMPs) in human serum [35]. AMPs have a key role in the stimulation of the immune response in cases of viral [36], bacterial [37] or fungal [38] infections by the induction of several cytokines and chemoattractants [36]. In humans, AMPs are secreted by different cells and are present in various body fluids such as saliva, serum, sweat, tears, nasal secretion, urine, cervicovaginal fluid, seminal fluid, etc. [35]. These AMPs may provide deeper insights into the molecular aspects of obesity and T2D; furthermore, they may also reveal potential therapeutic targets. The alterations in AMP abundance were investigated by other groups; the main finding of El-Mowafy’s group was the significant elevation of the α-defensin [39], while Vela et al.’s study showed a decrease in hepcidin levels in the sera of T2D patients compared with controls [40]. Interestingly, T2D patients with obesity also showed higher hepcidin levels compared with both non-obese T2D patients and controls [41]. These examples also illustrate the potential for closer investigation of AMPs in the future, but our knowledge concerning these proteins is still incomplete.

Another interesting aspect of the molecular background of obesity and T2D is related to the most studied post-translational modification (PTM) [42,43]: the phosphorylation carried out by protein kinases [42,43,44,45]. Dysregulation of kinase activity patterns might lead to the development of numerous diseases, such as cancers [46] and neurodegenerative diseases [47,48], but the development of T2D [49,50,51] was also reported. The investigation of phosphorylation patterns has great potential in obesity and T2D; it may provide profound insights into their pathological conditions and may result in the development of therapeutics [42,43,52,53]. Although serum-based investigations remain limited, numerous studies have analyzed the changes in phosphorylation patterns associated with T2D using cell cultures and tissue samples [49,50,51].

This study addresses an important gap in the field by performing comprehensive proteomics, as well as phosphoproteomics and kinase activation predictions on human serum samples. Furthermore, our study extends the investigation of proteoforms and AMPs, whose role in these diseases has not been explored extensively. Our aims also included the identification of enriched biological functions and pathways involved in obesity and T2D, thereby allowing a direct, closer investigation of their molecular bases.

## 2. Materials and Methods

All solvents and chemicals used in this study were of high purity and purchased from Sigma (St. Louis, MO, USA) unless otherwise stated.

### 2.1. Study Subjects and Sample Collection

The collection of the serum samples was approved by the Ethics Committee of the University of Debrecen (4845B-2017) and the National Institute of Pharmacy and Nutrition (OGYEI/2829/2017, date of approval: 31 January 2017). All participants provided written informed consent. Altogether, samples were collected from 134 individuals: 46 healthy volunteers, 43 obese patients and 46 patients with T2D mellitus. The relevant clinical data are available in Supplementary Table S1. The average age was 48.8 years in the Control group, the ratio between males and females was 1.1:1. In the Obesity group, the mean age was 50.9 years, the ratio between males and females was 1:1. In the T2D group, the average age was 51.2 years, and the ratio between males and females was 1.7:1.

Serum samples were depleted and used for DIA analyses from 134 individuals, including 45 healthy volunteers, 42 subjects with obesity and 47 patients having T2D. A total of 30 samples from each group (90 in total) were used for DDA-based proteomic and phosphoproteomic analyses.

### 2.2. Sample Preparation

The protein concentration of each serum sample was determined using a Pierce^TM^ BCA Protein Assay Kit (Thermo Scientific, Waltham, MA, USA), Microplate Procedure, based on the protocol provided by the manufacturer. The 14 most abundant serum proteins were depleted from samples for DIA analyses using High-Select^TM^ Top 14 Abundant Protein Depletion Mini Spin Columns (Thermo Scientific, Waltham, MA, USA). A total of 600 µg protein from each individual sample was subjected to depletion, and the procedure was performed according to the manufacturer’s protocol. Briefly, the sample-resin mixtures were gently homogenized, then, using an end-over-end mixer, incubation was performed for 10 min at room temperature. To retrieve the flow-through, we performed centrifugation for 2 min, 1000× *g*, at room temperature. The flow-throughs were dried in a speed-vac (Thermo Scientific, Waltham, MA, USA) until a final volume of 12.5 µL was obtained and used for the enzymatic digestion, performed with an iST kit (PreOmics GmbH, Martinsried, Germany) according to the manufacturer’s instructions. Briefly, to prepare the samples for the digestion, we added 12.5 µL of NHS-LYSE buffer to the samples, followed by incubation for 10 min, at 95 °C, 1000 rpm in a thermo shaker (Biosan, Riga, Latvia). For digestion, we added 50 µL of “DIGEST” solution, containing trypsin and Lys-C enzymes. The preparation of the “DIGEST” solution was based on the manufacturer’s protocol. The enzymatic digestion lasted for 2 h, at 37 °C, 500 rpm in a thermo shaker (Biosan, Riga, Latvia). Two hours later, we added 100 µL “STOP” reagent to inactivate the enzymes and to stop the digestion. In the next two steps, we purified the peptides using 200–200 µL “WASH-1” and “WASH-2” buffers; between the two steps, a short centrifugation was inserted (2 min, 2800× *g*, room temperature). To retrieve the purified peptides from the cartridge, we used the “ELUTE” buffer in two steps (2 × 100 µL), and the flow-through was kept in a clean tube. The eluted and purified peptides were dried in a speed-vac (Thermo Scientific, Waltham, MA, USA). The dried samples were redissolved in 100 µL 1% formic acid, and the corresponding pH was verified (pH 3–4). Pierce^TM^ C18 Tips (Thermo Scientific, Waltham, MA, USA) were equilibrated according to the manufacturer’s instructions, and after the binding of the peptides, a two-step elution was applied. First, 20% acetonitrile was used for the elution of less hydrophobic peptides, followed by an elution with 100% acetonitrile. The 20% and 100% acetonitrile-eluted samples were dried in a speed-vac (Thermo Scientific, Waltham, MA, USA) and stored at −20 °C until the LC-MS analysis.

In the case of tandem mass tag (TMT) labeling experiments, 120 µg of proteins were digested enzymatically from each sample on S-Trap^TM^ mini spin columns (ProtiFi, Fairport, NY, USA). Briefly, we added 23 µL 2X lysis buffer to the samples and clarified them from potential debris using a centrifugation step (8 min, 13,000× *g*, room temperature). Supernatants were removed and used for subsequent experiments. The reduction step was performed at 55 °C for 15 min using 2 µL 100 mM dithiothreitol, and then we alkylated the samples for 10 min at room temperature using 2 µL 200 mM iodoacetamide (BioRad, Hercules, CA, USA) solution. By adding 5 µL of 12% phosphoric acid (REANAL, Budapest, Hungary), we acidified the samples, inducing the precipitation of protein and promoting their efficient binding to the S-Trap^TM^ column. We added 350 µL of 100 mM triethylammonium bicarbonate/90% methanol buffer to each sample and transferred the mixtures onto S-Trap^TM^ mini columns, followed by centrifugation (30 s, 4000× *g*, room temperature). We then performed enzymatic digestion overnight at 37 °C using trypsin (Promega, Madison, WI, USA) at a 1:10 enzyme-to-protein ratio (1 µg/µL) in triethylammonium bicarbonate/LC-grade water (VWR Ltd., Radnor, PA, USA) (50 mM, pH 8.5) buffer. For peptide elution, we used 50 mM triethylammonium bicarbonate buffer, 0.2% formic acid and 50% acetonitrile solutions. Before isobaric labeling, the eluted peptides were completely dried using a speed-vac (Thermo Scientific, Waltham, MA, USA). Digested and vacuum-dried serum samples were dissolved in 120 µL triethylammonium bicarbonate (100 mM, pH 8.5) to a final concentration of 1 µg/µL.

From each dissolved sample, 11 µL was taken out and pooled to create a „supermix” used for the between-series normalization. From the 90 samples, ten 10-plex series were formed, each containing nine individual samples and one supermix. For isobaric labeling, 10 mass tags from a TMTpro 16plex Label Reagent Set (Thermo Scientific, Waltham, MA, USA) were used. Isobaric labeling was carried out following the vendor’s protocol. Right before the labeling procedure, we equilibrated the labeling reagents to room temperature one by one, then we redissolved the reagents in 220 µL anhydrous acetonitrile, followed by occasional vortexing for 5 min. After the preparation, we added 20 µL labeling reagent to 100 µL serum sample, which was followed by 1 h incubation at room temperature. The same procedure was repeated for the remaining 9 labels, except in the case of supermix, where we added 200 µL of labeling reagent to 990 µL of serum sample. The labeling in each case was quenched by the addition of 5 µL (50 µL for supermix) of 5% hydroxylamine (Thermo Scientific, Waltham, MA, USA). The 5% solution was prepared with LC-grade water (VWR Ltd., Radnor, PA, USA) using the factory provided 50% stock solution. This was followed by a 15 min incubation at room temperature. As a last step, samples and the supermix were mixed in a 1:1 ratio per series. Following this, 5% of each mixture was removed, dried and used for global proteomics measurements. First, a high pH reversed-phase fractionation was carried out using Pierce High pH Reversed-Phase Peptide Fractionation Kit (Thermo Scientific, Waltham, MA, USA) based on the protocol provided by the manufacturer. During fractionation, 8 fractions were collected using 8 different elution solutions for TMT-labeled peptides (Supplementary Table S2). The rest of the mixtures were used for phosphopeptide enrichment. Before the enrichment procedure, peptide samples were desalted using Sep-Pak C18 cartridges (Waters Corporation, Milford, MA, USA). The cartridges were conditioned with 5 mL of 50% acetonitrile/0.1% formic acid solution, followed by equilibration with 5 mL 0.1% formic acid. After redissolving the samples in 1 mL 0.1% formic acid, the peptide samples were slowly passed through the cartridges twice using clean syringes. Cartridges were washed with 5 mL 0.1% formic acid, and peptides were eluted with 1 mL 50% acetonitrile/0.1% formic acid solution. The eluted samples were completely dried in a speed-vac (Thermo Scientific, Waltham, MA, USA), then the phosphopeptide enrichment was carried out using a High-Select Fe-NTA Phosphopeptide Enrichment Kit (Thermo Scientific, Waltham, MA, USA) following the manufacturer’s guidelines. Briefly, peptide samples dried in a speed-vac were dissolved in 200 µL of Binding/Wash buffer. Fe-NTA columns were equilibrated using 2 × 200 µL of Binding/Wash buffer, with centrifugation (30 s, 1000× *g*, room temperature) applied between the equilibration steps. Samples were then loaded onto the equilibrated Fe-NTA columns and incubated at room temperature for 30 min. During the incubation, the columns were gently agitated every 10 min to keep the resin in suspension. After 30 min, the columns were centrifuged (30 s, 1000× *g*, room temperature), and the flow-throughs were discarded. The columns were washed with 3 × 200 µL of Binding/Wash buffer and once with 200 µL of LC-grade water (VWR Ltd., Radnor, PA, USA). Between washes, the columns were centrifuged at 1000× *g* for 30 s at room temperature. Phosphopeptides were eluted using 2 × 100 µL of Elution buffer. Eluates were collected into clean tubes via centrifugation at 1000× *g* for 30 s at room temperature. Eluates were pooled, acidified using concentrated trifluoroacetic acid and dried down.

### 2.3. LC-MS Analyses

All samples prepared for DIA analyses were redissolved in 20 µL 1% formic acid solution. Before the LC-MS analysis, samples were spiked with iRT peptides (Biognosys AG, Zurich, Switzerland) according to the manufacturer’s protocol. The LC-MS analyses were performed on an Easy-nLC 1200 (Thermo Scientific, Waltham, MA, USA) coupled to an Orbitrap Fusion mass spectrometer (Thermo Scientific, Waltham, MA, USA). The spiked samples were loaded on to an ACQUITY UPLC M-Class Symmetry C18 Trap column (180 µm × 20 mm, 100 Å pore size, 5 µm particle size; Waters Corporation, Milford, MA, USA) and further separated on a nanoEase M/Z Peptide BEH C18 analytical column (75 µm × 150 mm, 130 Å pore size, 1.7 µm particle size; Waters Corporation, Milford, MA, USA). For the separation of the peptides, a 50 min gradient elution was performed. Solvent A was 0.1% formic acid in LC-MS grade water, while solvent B was 95% acetonitrile containing 0.1% formic acid. The flow rate was set to 300 nL/min. Mass spectrometry analyses were performed in positive ion mode using an NSI ion source. The spray voltage was 2300 V, the sweep gas was 0.2 L/min, and the temperature of the transfer capillary was 300 °C. For MS1 scans, we used an Orbitrap mass analyzer; the scan range was between 350–1650 *m*/*z*. The resolution was set to 120,000, the AGC target value was 1 × 10^6^, and MS1 spectra were recorded in profile mode. After MS1, we used 27% HCD collision energy for ion fragmentation, with isolation in a Quadrupole. For MS2, the scan range was between 200–2000 *m*/*z* and the MS2 spectra were recorded in centroid mode. As a detector, we used Orbitrap; the resolution was set to 30,000, and the AGC target value was set to 1 × 10^6^. The data was collected using the DIA method, with 33 overlapping scan windows.

The TMT-labeled samples were analyzed using an Orbitrap Fusion Lumos Tribrid mass spectrometer on-line coupled to a Thermo Ultimate 3000 nanoHPLC (Thermo Scientific, Waltham, MA, USA). The labeled samples were loaded onto an Acclaim^TM^ PepMap^TM^ 100 C18 trap column (0.3 mm × 5 mm, 100 Å pore size, 5 µm particle size; Thermo Scientific, Waltham, MA, USA) and further separated on a nanoEase M/Z Peptide BEH C18 analytical column (75 µm × 250 mm, 130 Å pore size, 1.7 µm particle size; Waters Corporation, Milford, MA, USA). For the global proteomics and the phosphoproteomics measurements, different LC gradient lengths were used, 56 min and 88 min, respectively. For the MS analysis, data were acquired in a DDA fashion in positive ion mode. For peptide and protein identification, MS2 ion trap CID (35% collision energy) spectra were recorded. For MS2, a Quadrupole was used for isolation; the scan range was between 570–1500 *m*/*z*. The AGC target value was 50%, and spectra were recorded in centroid mode. For quantification, SPS MS3 HCD (55% collision energy) spectra were recorded in the Orbitrap (R = 50,000) by isolating the six most abundant fragment ions generated during the MS2 ion trap CID event and fragmenting them in the ion routing multipole using HCD activation. The scan range was between *m*/*z* 100–500, AGC target value was set to 200% and the spectra were recorded in centroid mode.

More than 30 subjects (biological replicates) for each group were included in the study and no technical replicates were run.

### 2.4. Data Analysis

For DIA data analysis, we used DIA-NN (v1.8.1) [54]; the DIA raw data were aligned against a human subset of the Swiss-Prot database [55] (downloaded November 2022). The following settings were applied: FASTA digest for library-free search, missed cleavages were set to 2, the maximum number of variable modifications was 2, methionine oxidation and N-terminal acetylation were added, and specific cysteine modification (+113.083 Da) was added as a fixed modification.

The results of DDA experiments were analyzed using Proteome Discoverer (v3.1) software (Thermo Scientific, Waltham, MA, USA). The human subset of the Swiss-Prot database [55] was used to align the raw data (downloaded January 2024). Missed cleavages were set to 2, while the maximum number of dynamic modifications was set to 4 per peptide. As a static modification, cysteine carbamidomethylation was added, as well as TMTpro on any N-termini and lysine. For dynamic modification, N-terminal acetylation, methionine oxidation, as well as phosphorylation on serine, tyrosine and threonine, were set.

Those proteins that were accepted were identified with at least two peptides. The Mann–Whitney U test was performed using IBM SPSS Statistics for Windows, version 23.0 (IBM Corp., Armonk, NY, USA), followed by the Benjamini–Hochberg method for the assessment of false discovery rate (FDR) with GraphPad Prism (v8.0.1) [56]. Changes were considered statistically significant with a *p*-value of <0.05 and an FDR of <1%.

### 2.5. Network Analysis

The differentially abundant proteins were used for network generation using the STRING DB (v11.5) [57]. For the interaction score, a high confidence (0.9) was set. Due to the limited number of DAPs, we included the max. 50 first shell interactors. Proteins without any detected interactions were removed from the networks to improve the clarity and interpretability of subsequent analyses. The created networks were imported into the Cytoscape (v3.10.2) [58] software. In order to better understand the molecular background of these conditions, we performed gene ontology (GO) analyses by using the clueGO (v2.5.10) [59], similarly to the previous study of our workgroup [60]. CytoHubba (v0.1) [61] was used to map the top 10 hub proteins from each network based on degree.

### 2.6. Examination of AMPs

The proteins identified in our analyses were checked to see if they are AMPs. As a reference, we used the list of AMPs summarized in the study by Kalló et al. [35] We supplemented the list of identified AMPs in our DIA or DDA-based proteomics experiments with AMPs identified in our workgroup’s previously published Olink proteomics dataset [62]. Proteins that showed a statistical difference at *p* < 0.05 were selected for network and GO analyses. For Olink data, statistical analyses were performed by the authors. Proteins without any detected interactions were removed from the networks to improve the clarity and interpretability of subsequent analyses.

### 2.7. Ingenuity Pathway Analyis^®^

We submitted our data to the IPA platform (QIAGEN Inc., Redwood City, CA, USA) to derive additional bioinformatics annotations along with potential protein interaction networks, canonical pathways, as well as associated biological functions and processes [63]. To organize the results, we relied on *p*-values from right-tailed Fisher’s exact tests and z-scores for activation or inhibition of canonical pathways, which were calculated by the software’s own algorithms.

### 2.8. Kinome Analysis

The kinase prediction analysis from the identified phosphopeptides was done using PhosphoSitePlus (v6.7.4) [64] with default settings. Peptides with multiple phosphorylations were subjected to individual prediction analysis.

## 3. Results

With this study, we aimed to gain deeper insights into the pathophysiology of obesity and T2D by revealing differentially abundant proteins (DAPs) and AMPs using unbiased LC-MS-based omics of serum, which allowed for the identification of pathways and protein–protein interaction (PPI) networks affected by obesity and/or T2D. Furthermore, we extended our study with phosphopeptide enrichment and kinase activity prediction analysis to elucidate how this PTM was altered under these pathological conditions.

### 3.1. Proteomics Analysis of Serum Samples

**Figure 1:**
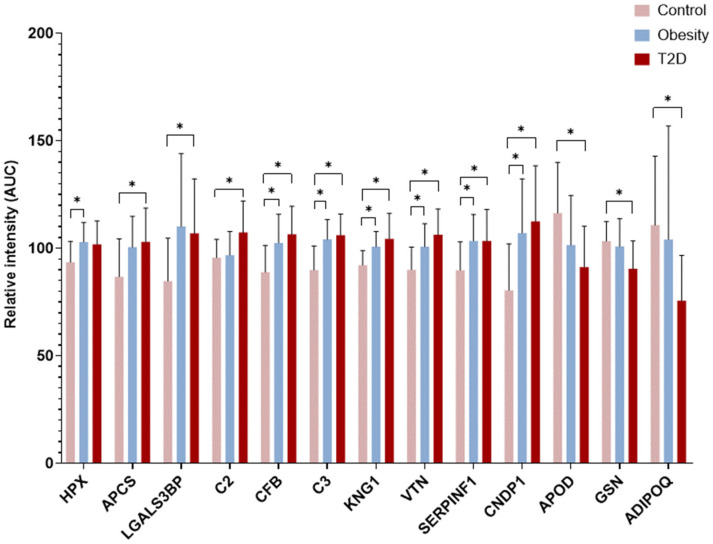
DAPs after Mann–Whitney U-tests followed by FDR correction (*: q < 0.01) in TMT labeled samples. The figure shows the mean values of the relative intensities of DAPs and their standard deviations. Pink colored bars represent the Control group, blue bars indicate the Obesity group, while red bars designate the T2D group.

In our analyses, we applied two proteomics strategies (Supplementary Figure S1). In the first workflow, TMT labeling and DDA-based LC–MS/MS were carried out, and 30 samples per group were examined. In the 90 samples, 235 proteins were identified (Supplementary Table S3). The statistical analysis showed 13 DAPs with statistically significant differences among the examined groups. The following seven proteins are present in higher amounts in the Obesity group compared with the Control group (Figure 1): hemopexin—HPX, complement factor B—CFB, complement C3—C3, kininogen-1—KNG1, vitronectin—VTN, pigment epithelium-derived factor—SERPINF1 and beta-Ala-His dipeptidase—CNDP1. When the T2D group was compared to controls, nine proteins (serum amyloid P-component—APCS, galectin-3-binding protein—LGALS3BP, complement C2—C2, CFB, C3, KNG1, VTN, SERPINF1 and CNDP1) were present in higher amounts, and three proteins (apolipoprotein D—APOD, gelsolin—GSN and adiponectin—ADIPOQ) in lower amounts. In the comparison of the Obesity and T2D groups, we could not identify any statistically significant changes in protein abundances.

Along with the TMT labeling, a label-free quantification applying DIA was also carried out. Samples were depleted to decrease the amount of 14 highly abundant serum proteins. We identified 232 proteins in 134 depleted serum samples (Supplementary Table S4). After FDR correction, none of the proteins showed statistically significant differences.

#### 3.1.1. Network Analysis Highlights the Similarities in the Molecular Background of Obesity and T2D

**Figure 2:**
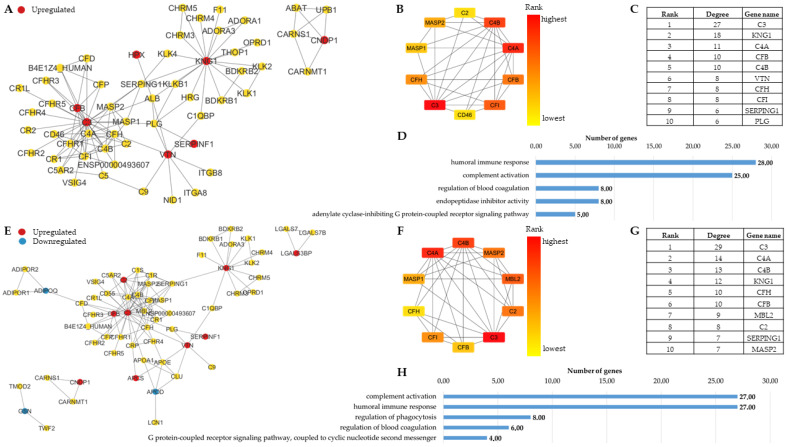
STRING network and functional GO analysis of DAPs associated with obesity and T2D in human serum samples, respectively. Network of DAPs in Obesity versus Control (**A**), and T2D versus Control (**E**). The dots indicate proteins (DAPs and their max. 50 first shell interactors), and the lines the interactions between them. Proteins showing statistically significant changes compared with controls (q < 0.01) are indicated as red or blue dots, red for increased, while blue for decreased amount in the group studied. Networks of the top 10 hub proteins in obesity (**B**) and T2D (**F**), along with proteins with the highest degree value for obesity (**C**) and T2D (**G**), respectively, are shown. The top 5 GO functions enriched in obesity (**D**) and T2D (**H**) are indicated.

The PPI networks characteristic of obesity or T2D were generated using STRING (Figure 2). To examine the enriched biological functions involved in the development of obesity and T2D, we also performed gene ontology (GO) analysis.

The network characteristic of obesity (Figure 2A) contains three clusters of proteins related to the complement cascade and its regulation, cell–cell connections in relation to blood coagulation, as well as signaling pathways regulated by G proteins. In a separate cluster not related to the main network, we could identify proteins involved in carnosine metabolism. The proteins with the highest number of interactions (hubs) (Figure 2B,C) participate in the complement cascade and blood coagulation, indicating their key role in obesity. These results are consistent with the enriched biological functions identified from the DAPs in obesity (Figure 2D), highlighting the significance of humoral immune response, blood coagulation and adenylate cyclase signaling in obesity.

The network characteristic of T2D (Figure 2E) was very similar to the one observed in obesity, containing a core network with three clusters. Proteins involved in carnosine metabolism and cell adhesion were also present as separate clusters. The hub proteins (Figure 2F,G) and enriched functions (Figure 2H) were very similar in the two networks, indicating that similar biochemical functions were altered in both conditions. Compared with obesity, the regulation of phagocytosis appeared as a novel enriched biological function in T2D (Figure 2H).

#### 3.1.2. Canonical Pathways Characteristic of Obesity and T2D Show the Differential Involvement of Lipid Metabolism and Atherosclerosis Signaling in T2D

**Figure 3:**
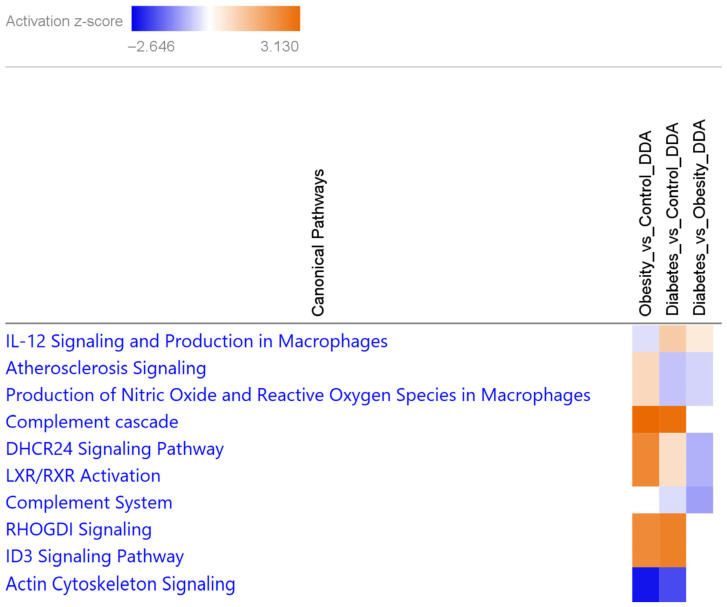
Heatmap of the top 10 canonical pathways characteristic of obesity or T2D from DAPs by IPA. Increased and decreased pathways are represented as orange or blue boxes, respectively, based on their activation z-score (scale on top). Canonical pathways with no activation are indicated with white boxes.

To get more information on the pathways with a role in obesity and/or T2D, we performed Ingenuity Pathway Analysis^®^ (IPA) (Figure 3, Supplementary Figure S2). Reinforcing STRING and GO analyses summarized in Figure 2, the complement cascade was activated in both pathological conditions, while a considerable difference was observed regarding Liver X Receptor/Retinoid X Receptor (LXR/RXR) activation and Delta(24)-sterol reductase (DHCR24) signaling, with less activity in T2D compared with obesity for both affected canonical pathways. The atherosclerosis signaling, as well as the production of nitric oxide (NO), was regulated in opposite directions with elevation in obesity and decrease in T2D.

### 3.2. AMPs Indicate the Common Proteomic Landscape of Lipid Metabolism and Immune Regulation in Obesity and T2D

**Figure 4:**
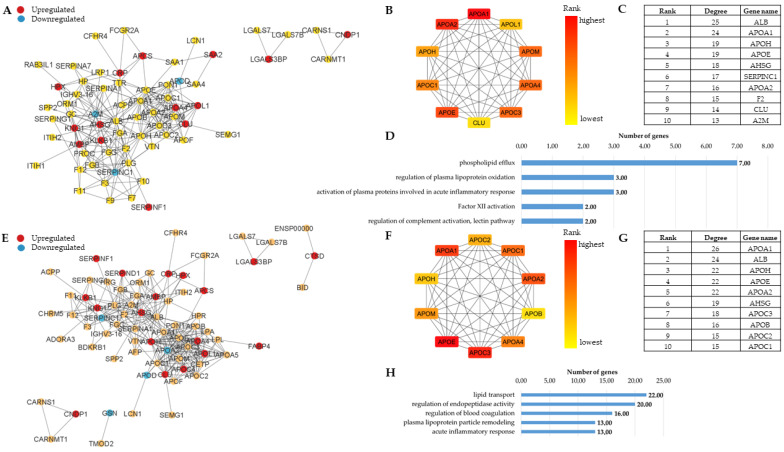
Network and functional analysis of AMPs identified in obesity and T2D, respectively. Network of AMPs in Obesity versus Control (**A**), and T2D versus Control (**E**). The dots indicate proteins (AMPs and their 50 first shell interactors), and the lines indicate the interactions between them. Proteins showing statistically significant changes compared with controls (*p* < 0.05) are indicated as red or blue dots, red for increased, while blue for decreased amount in the studied group. Networks of the top 10 hub proteins in obesity (**B**) and T2D (**F**), along with proteins with the highest degree value for obesity (**C**) and T2D (**G**), respectively, are shown. The top 5 GO functions enriched in obesity (**D**) and T2D (**H**) are indicated.

Differentially abundant AMPs from our DIA and TMT experiments (Supplementary Table S5) were supplemented with our previous Olink data [62] to generate PPI networks (Figure 4). A core network with two clusters and two separate small clusters was observed in the case of obesity (Figure 4A). One of the core clusters contains several upregulated proteins having a role in heme homeostasis, antioxidant activity and the initiation of blood coagulation, while two downregulated proteins were involved in protease inhibition. The other cluster contained mainly apolipoproteins, which are involved in lipoprotein homeostasis. They have a central place in the network, many of them being among the top 10 proteins with the highest number of interactions (Figure 4B,C). The enriched biological functions (Figure 4D) highlighted the importance of these proteins. Specifically, they took part in lipid metabolism-related functions and functions engaged in inflammatory responses and complement activation.

Compared with obesity, we found more differentially abundant AMPs in T2D, and they were present in two clusters and several small clusters not linked to the core network (Figure 4E). The structure of the network, as well as the top 10 hub proteins (Figure 4F,G) and the GO functions (Figure 4H), were very similar to those observed in the case of obesity. Regarding the GO functions, however, the ranks were different. While, in obesity, the phospholipid efflux was the most prominent, the regulation of the endopeptidase activity proved to be the most enriched function upon T2D.

### 3.3. Phosphoproteomics Analysis of Non-Depleted Human Serum Samples Complements the Proteomics Results and Identifies Further Functions and Proteins Characteristic to Obesity or T2D

A phosphopeptide enrichment analysis was carried out using non-depleted TMT-labeled samples, and the results were summarized in Supplementary Table S6. We identified 63 phosphopeptides belonging to 42 proteins. We also performed peptide-level analyses, and we were unable to detect non-phosphorylated counterparts for 43 phosphorylated peptides (Supplementary Table S6).

**Figure 5:**
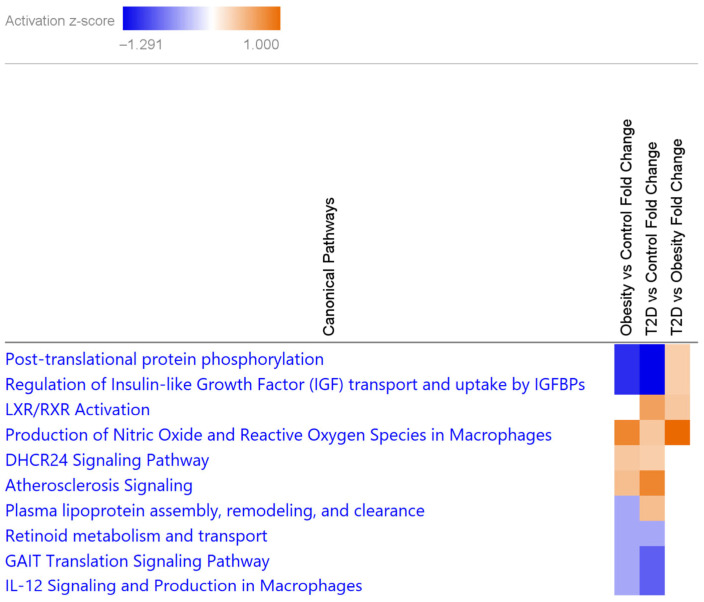
Heatmap of the top 10 canonical pathways based on serum phosphoproteomic characteristics to obesity or T2D by IPA. Increased and decreased pathways are represented as orange or blue boxes, respectively, based on their activation z-score (scale on top). Canonical pathways with no activation are indicated with white boxes.

Throughout our data analyses, we identified 30 differentially abundant phosphoproteins and 41 differentially abundant phosphopeptides, which were further subjected to IPA analyses (Supplementary Table S6). IPA networks from the phosphoproteome showed relevance to highly similar canonical pathways, such as those related to lipoprotein and cholesterol metabolism, hypertriglyceridemia, weight gain and to the quantity of insulin in the blood, to be important in both conditions (Supplementary Figure S3). Some of the top canonical pathways identified at the proteome level (e.g., atherosclerosis signaling, LXR/RXR activation, DHCR24 signaling and production of NO) were also present at the phosphoproteome level, indicating their regulation by phosphorylation (Figure 5).

As expected, post-translational protein modification by phosphorylation was the canonical pathway with the highest change, followed by regulation of insulin-like growth factor (IGF) transport and uptake of IGFBPs with the same scores. A more pronounced phosphorylation is observed in the T2D group in the case of the above-mentioned two pathways, the DHCR24 signaling, atherosclerosis signaling, response to elevated platelet activation and LXR/RXR activation pathways, while higher phosphorylation in obesity was characteristic of the production of NO, IL-12 signaling and GAIT translation signaling.

The top 5 kinase enzyme families based on their phosphosite binding probability were retrieved from databases. Our data suggest that, in obesity, the activity of CK2 and AGC is increased, and of NKF1 is decreased (Supplementary Table S6), whereas no major differences in phosphorylation patterns and changes in kinase activities were observed in T2D.

## 4. Discussion

Obesity, often driven by harmful habits such as poor diet and a sedentary lifestyle, is a major risk factor for the development of T2D [1,2,6,7,8]. As a consequence of an excessively high caloric intake and lack of physical activity, the body responds by storing energy in the form of triglycerides within adipocytes, which leads to hypertrophy and hyperplasia of adipose tissue over time [65]. The rapid occurrence of these processes can lead to hypoxia and cell death in the tissue, which in turn is a known trigger for the initiation of inflammation in adipose and other insulin-sensitive tissues (muscle, liver) [65,66,67]. The mechanism of chronic low-grade inflammation in adipose tissue is well investigated; briefly, the aforementioned processes result in the infiltration and transformation of macrophages (M2 to M1) that secrete several kinds of pro-inflammatory cytokines, e.g., interleukin-6 (IL-6) and tumor necrosis factor alpha (TNF-α), known to play a fundamental role in the development of insulin resistance (IR) [66,68]. Beyond these cytokines, additional markers of inflammation have been reported with markedly elevated levels in obesity and T2D. Several elements of the complement cascade are secreted by the adipose tissue, such as C3, which serves as the central player in the cascade [68,69,70,71]. Besides the elimination of pathogens, the role of the complement system is also crucial in cell metabolism [68,69,72]. The relationship between elevated C3 levels and IR has been reported [73]. In adipocytes, its desarginated cleavage product C3a-desArg, also known as acylation-stimulating protein (ASP), not only exerts insulin-like activity but also promotes the influx of pro-inflammatory cytokines, thereby contributing to IR [68,71,72]. These inflammatory processes induced by elevated C3 levels further impair insulin secretion in pancreatic β-cells, ultimately leading to T2D [69], with pathological sequelae studied by several research groups [68,74,75].

Parallel to these biological processes, the increased levels of pro-inflammatory cytokines reduce ADIPOQ secretion, contributing to the development of T2D [76,77,78]. The relationship between C3 and ADIPOQ can be considered close, as they have a significant influence on the development and preservation of persistent, chronic, systemic inflammation. While ADIPOQ is known to have an anti-inflammatory effect by promoting polarization of macrophages to M2 type [79], an increase in C3 levels and a decrease in ADIPOQ levels promote inflammation [80]. Our study was able to demonstrate these changes at the proteome level in the serum, thereby confirming the background of the activation of inflammatory processes. Furthermore, our network and functional analyses also supported the central role of the complement system and the humoral immunity in the pathophysiology of obesity and T2D.

In addition to the complement cascade components, we also revealed an increase in proteins that play an important role in the regulation of blood coagulation. KNG1 is known to induce blood coagulation through contact activation, but it also leads to the formation of bradykinin, which results in the release of inflammatory mediators [81,82]. On the other hand, due to endothelial dysfunction developing in obesity and T2D, its pro-coagulant effect prevails, thus playing an important role in the development of cardiovascular events, which are considered common complications to both pathological conditions [83,84]. Another protein with a dual role is VTN, which is known to play a role in the generation of inflammatory processes through the complement system [85]. Similar to KNG1, VTN can also promote thrombotic events by stabilizing plasminogen activator inhibitor 1 (PAI-1) [86]. The relevance of these proteins is highlighted by their organization into well-defined clusters within the network of DAPs common to obesity and T2D, which are closely associated with the complement system proteins. In addition, our GO analyses also confirmed that these two biological processes are closely linked in obesity and T2D.

An interesting finding is the elevated level of the HPX. Although its exact role in the human body is controversial, HPX’s main function is to transport heme to the liver for degradation [87,88], but it is also known as an acute phase protein [87,88,89]. Other research groups have already investigated HPX levels in various experimental models, but the results to date are contradictory overall [78,89,90,91]. In our study, we observed elevated levels of HPX in human serum samples in both pathological conditions. This finding is reasonable, as HPX has been reported to correlate positively with serum triglyceride levels [90], elevated BMI [92] and red blood cell damage [93]. The connection of HPX to the complement system may provide another explanation, although the evidence is contradictory [91]. However, studies focusing on the relationship between the complement cascade and HPX are limited; a few studies suggest that hemolysis can activate the complement system [88,94,95,96].

We observed an increase in the amount of SERPINF1, a protein known to be secreted by adipocytes, in both obesity and T2D. Although it is not a classic serine protease inhibitor, SERPINF1 plays an important role in certain biochemical mechanisms, such as metabolic inflammation, where free fatty acids released through lipolysis can activate signaling pathways that lead to the release of inflammatory cytokines (IL-6) and eventually to IR [97]. Some studies have shown that a reduction in adipose tissue mass through surgery or exercise significantly decreased the levels of both SERPINF1 and inflammatory marker levels [98,99]. Overall, we were able to detect elevated levels of proteins with diverse biological functions in serum samples from obese and T2D individuals using a shotgun proteomics method. These proteins are closely related to each other, as illustrated by the networks of DAPs.

Some of the proteins identified in our study, besides their well-known functions, have antimicrobial activity and also operate as AMPs. Many proteins associated with lipoprotein metabolism and blood coagulation fall into this category. CRP is a well-known marker of systemic inflammation, and apolipoprotein A4 (APOA4) and apolipoprotein L1 (APOL1) regulate lipid and lipoprotein particle metabolism. Their antimicrobial activity has also been described [100,101,102]. HPX contributes to defense by neutralizing heme [96,103]. It was shown that increased SERPINF1 levels may be associated with the extension of inflammatory processes, as it may contribute to elevated IL-6, which plays a central role in maintaining chronic, low-grade inflammation [97]. Also remarkable is FABP4, which, as a lipid-binding protein, plays a key role in regulating metabolism, but, similar to SERPINF1, its levels correlate positively with both IL-6 and CRP levels [104]. In addition to elevated levels of the above-mentioned AMPs, we observed decreased levels of anti-inflammatory AMPs. APOD and alpha-2-macroglobulin (A2M) are known to contribute to the control of oxidative stress and inflammation, and their reduction helps to maintain the inflammation [101,104,105]. The network of AMPs observed in our study shows high similarity between obesity and T2D, and links antimicrobial defense, lipid metabolism and coagulation processes, showing similar underlying mechanisms in the case of obesity and T2D.

The results of network-level analyses, both in the case of DAPs and AMPs, show highly similar networks in the cases of obesity and T2D, indicating that the two clinically distinct pathological conditions share considerable similarity at the molecular level.

Protein phosphorylation is one of the most studied PTMs [42,43], occurring on serine, threonine or tyrosine residues in mammalian organisms [43]. Phosphorylation has a key role in physiological signal transduction and cell differentiation, making it a fundamental process for the regulation of various biological functions. Dysregulated phosphorylation can contribute to the development of a variety of diseases, as evidenced by numerous studies highlighting altered phosphorylation patterns in cancer [46], neurodegenerative diseases [47,48] and T2D [49,50,51].

To date, numerous studies have investigated signaling pathways associated with T2D; however, most of the findings are derived from cell cultures or tissue samples. Thimmappa et al. [49] have found that specific kinases such as NTRK1, SYK and PRKACA are activated in neutrophils extracted from blood samples following high glucose treatment. The targets of these kinases are involved in the Rho GTPase signaling pathway, leading to neutrophil activation and an altered immune response to infections. Another study conducted by Nunez Lopez et al. [51] studied circulating extracellular vesicles from human serum samples and identified several phosphorylated kinases that are upregulated in the development and progression of T2D, including AKT1, GSK3B, LYN, MAP2K2, MYLK and PRKCD. Moreover, they found that CDK1 and PKCδ may play a critical role in the obesity-to-T2D progression, with elevated levels of these kinases contributing to reduced insulin secretion capacity and increased IR. Based on our kinome prediction analyses, we observed increased activity of casein kinase 2 (CK2) in obesity. CK2 is a well-known serine/threonine kinase, with several fundamental roles in inflammation, cell growth and metabolism [106]. Enhanced activity of CK2 has been reported from human adipocytes [107], highlighting the involvement in obesity-related pathologies, further supported by animal studies [108,109]. Increased activity of several members of the AGC kinase family [110] also has an important influence on maintaining cell homeostasis and metabolism, promoting adipogenesis and the development of obesity [111,112]. Interestingly, our data also indicated the reduced activity of members of the new kinase family 1 (NKF1). Protective effects of NKF1 family kinases, e.g., SH3 domain binding kinase family member 1 (SBK1) [113], have been reported earlier [114]; however, the same study also showed increased lipid accumulation in mouse hepatocytes lacking NKF1 family kinases [114]. Another group of kinases showing reduced activity was the CMGC (cyclin-dependent kinases, mitogen-activated protein kinases, glycogen synthase kinases and CDK-like kinases [115]) family. Similar to the NKF1 family, reduced CMGC kinase activity has been associated with lipid accumulation [116,117]. These findings highlight the relevance of kinome analyses in elucidating the molecular mechanisms of obesity and T2D.

Many of the canonical pathways were characteristic of either obesity or T2D, indicating regulation by phosphorylation based on our phosphoproteomics results. While the tendency in the global and phosphoproteomics was similar regarding NO production, it was the opposite with atherosclerosis signaling, LXR/RXR activation, DHCR24 signaling and IL-12 signaling, further highlighting the importance of phosphoproteomics in the study of obesity and T2D [84,118,119,120].

Considering that the disease-characteristic pathways regulated by phosphorylation may contain druggable components, we searched the literature for already-used therapeutic targets and/or drugs targeting the components of these pathways. The regulation of insulin-like growth factor (IGF) transport and uptake by the IGFBPs pathway plays an essential role in maintaining cellular metabolic balance, and its regulation through phosphorylation is well-documented [121]. Deviations in its phosphorylation pattern can disrupt metabolic homeostasis, yet no targeted therapeutic intervention currently exists to correct such alterations. Despite its central role in lipid metabolism and inflammation, no direct therapeutic agent is available that can specifically modulate phosphorylation-dependent regulation within the LXR/RXR pathway. The RXR agonist bexarotene is used clinically for cutaneous T-cell lymphoma, although its application is limited by hyperlipidemia as a side effect [122]. Experimental RXR agonists, such as LG268 (LG100268), have shown promising effects on appetite suppression and inflammation *in vivo* [123]. Similarly, the well-known LXR agonist T0901317 influences lipid homeostasis but induces lipogenesis [124], preventing its progression to clinical trials.

Nitric oxide (NO) and reactive oxygen species (ROS) are produced in macrophages in response to inflammatory cues through complex phosphorylation-driven signaling pathways, maintaining a persistent inflammatory microenvironment [125,126]. Several candidate molecules have been explored to mitigate this process and act on the production of nitric oxide and reactive oxygen species in the macrophage pathway. One notable example is ruboxistaurin (LY-333531), a PKCβ inhibitor that modulates phosphorylation events and has promising effects in reducing inflammation [127]. Regarding the DHCR24 signaling pathway, no direct pharmacological inhibitor is currently available in clinical practice. However, *in vitro* studies suggest that sitagliptin, widely used in the treatment of T2D, may lower DHCR24 levels [128], implying a potential indirect regulatory effect.

Atherosclerosis is driven by a complex network of molecular events, including phosphorylation-mediated regulation. Elevated phosphorylation of p38 MAPK has been documented in T2D [129], highlighting the involvement of atherosclerosis signaling in disease progression. Losmapimod, a p38 inhibitor, has been developed with the aim of targeting this pathway [130].

A recent study suggests that chronic metabolic stress, such as that found in obesity and T2D, may also promote the formation of phosphorylation-driven epichaperome complexes. According to the publication, these stable HSP90/HSP70-based structures depend on specific serine phosphorylation events (at Ser226 and Ser255) and are developed under prolonged stress [131]. Considering the altered kinase activities identified in our analyses, the persistent inflammation and oxidative stress observed in metabolic diseases may presumably promote the occurrence of epichaperome phosphorylation, although we did not get evidence for it in our dataset. This phosphorylation-dependent proteoform regulation may also contribute to the altered signaling pathways in obesity and T2D and may raise further questions for future research.

At the same time, this finding emphasizes the importance of proteoforms in studying complex diseases, as information about different proteoforms can reflect different biologically important details that can help us better understand the pathomechanisms of various diseases, such as obesity and T2D.

We are aware that our study has some limitations: the subjects involved came from the same geographical region, and although the mean age of the recruited subjects was similar, the age span between the youngest and oldest subjects was almost 30 years. Due to the relatively limited number of recruited subjects, the influence of age and gender was not examined in detail. Another limitation of our study is that we utilized only one sample type: blood.

## 5. Conclusions

Our study applied a comprehensive and integrative proteomic strategy on human serum samples, combining DIA and DDA experiments with phosphopeptide enrichment, as well as network and pathway analyses. Unlike many previous investigations that relied on animal or *in vitro* models, the data in this study were derived directly from human specimens, increasing the clinical relevance and translational potential of our findings. Importantly, while network analyses show that obesity and T2D share many similarities, our phosphoproteomic data uncovered distinct phosphorylation patterns between the two conditions, suggesting that molecular alterations at the level of proteoforms may play a critical role in disease progression. At the same time, our data can provide druggable pathways with possible utilization in the management of obesity and T2D.

## Data Availability

Published data are available within the article or in Supplementary Materials. Metadata regarding the experiments are presented in .sdrf format, created by lesSDRF [132]. The mass spectrometry proteomics data have been deposited to the ProteomeXchange Consortium via the PRIDE [133] partner repository with the dataset identifiers repository under the identifiers PXD058428, PXD058424, and PXD058421.

## Supplementary Materials

The following supporting information can be downloaded at: https://www.mdpi.com/article/10.3390/proteomes13040067/s1, Supplementary Figure S1: applied workflow for sample preparation and data analysis; Supplementary Figure S2: protein–protein interaction networks (PPI) of identified proteins (DDA) generated by Ingenuity Pathway Analysis^®^ (IPA); Supplementary Figure S3: PPI and canonical pathway analyses of identified phosphoproteins (DDA) generated by IPA; Supplementary Table S1: Patient data. Disease groups, age, sex, diabetes length in years, BMI values, and sample utilization for DDA or DIA experiments are indicated. “NA” is for not applicable, since no data are available; Supplementary Table S2: applied solutions for high-pH fractionation of TMT-labeled samples; Supplementary Table S3: Identified TMT-labeled proteins (DDA) with their quantitative values. UniProt identifiers and gene names of proteins are indicated; Supplementary Table S4: identified label-free proteins (DIA) with their quantitative values. UniProt identifiers and gene names of proteins are indicated; Supplementary Table S5: Identified AMPs from DDA, DIA and Olink experiments, with their up- and down-regulation patterns. UniProt identifiers and gene names of proteins are indicated; Supplementary Table S6: identified TMT-labeled phosphopeptides (DDA) with their phosphorylation sites, quantitative values and kinome analyses. UniProt identifiers and gene names of proteins are indicated.
