# Supplementary material for: Comprehensive Insights into Obesity and Type 2 Diabetes from Protein Network, Canonical Pathway, Phosphorylation and Antimicrobial Peptide Signatures of Human Serum
Source: Proteomes. 2025 Dec 17;13(4):67. doi: 10.3390/proteomes13040067 (PMC12736859; doi:10.3390/proteomes13040067)
Supplement: Supplementary file 1 [file proteomes-13-00067-s001.zip › Supplementary_Figure_S2.pdf]

Supplementary Figure S2. Protein-protein interaction networks of identified proteins generated by Ingenuity Pathway Analysis®.

Obesity versus Control (Network 1). Network 1 : Obesity_vs_Control_DDA : UDebrecen_Serum_DDA_12_4_24 : OBESITY_to_Control_log2FC

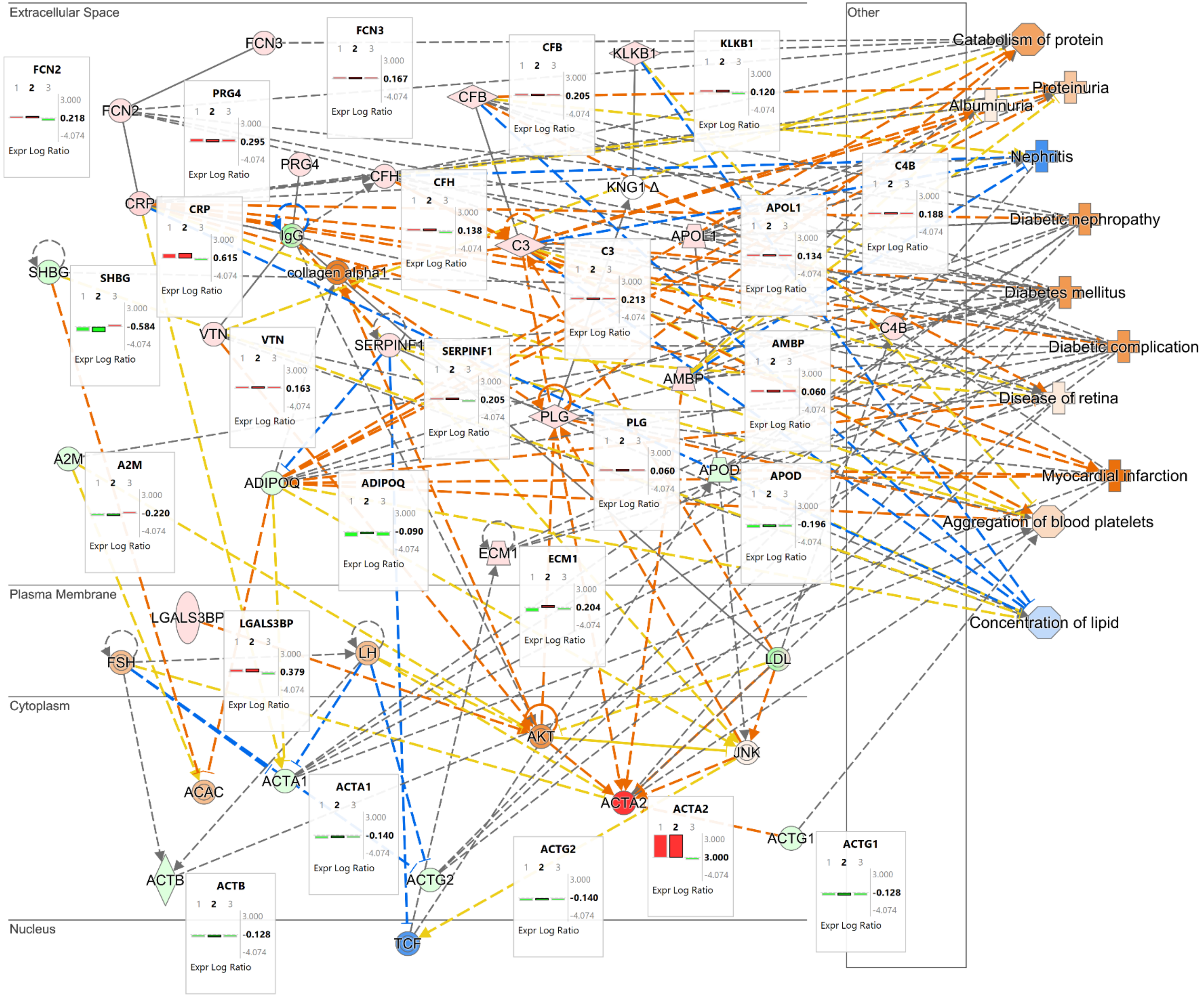

© 2000-2025 QIAGEN. All rights reserved.

Obesity versus Control (Network 2).

Network 2 : Obesity_vs_Control_DDA : UDebrecen_Serum_DDA_12_4_24 : OBESITY_to_Control_log2FC

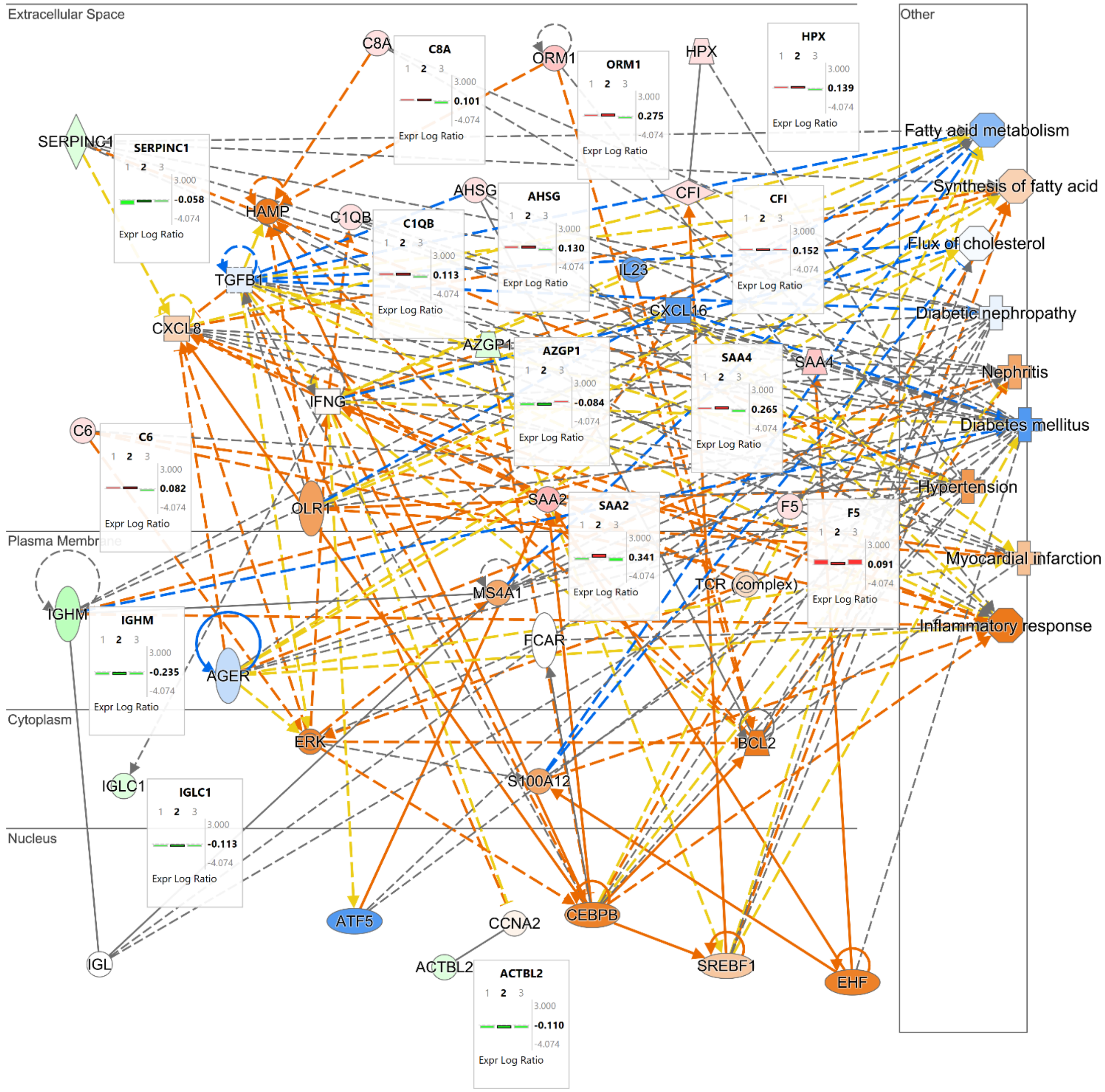

© 2000-2025 QIAGEN. All rights reserved.

T2D versus Control (Network 1).

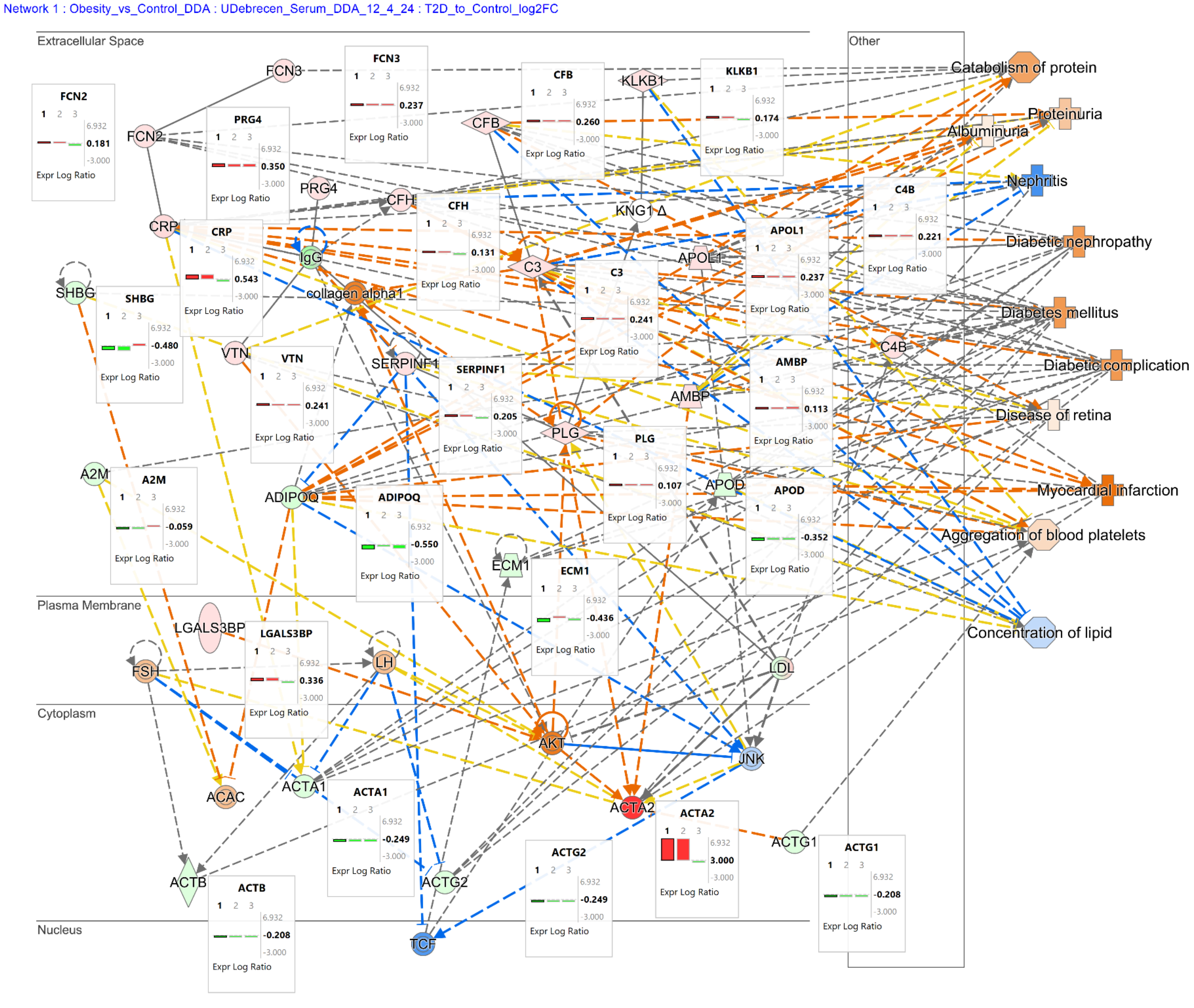

© 2000-2025 QIAGEN. All rights reserved.

T2D versus Control (Network 2).

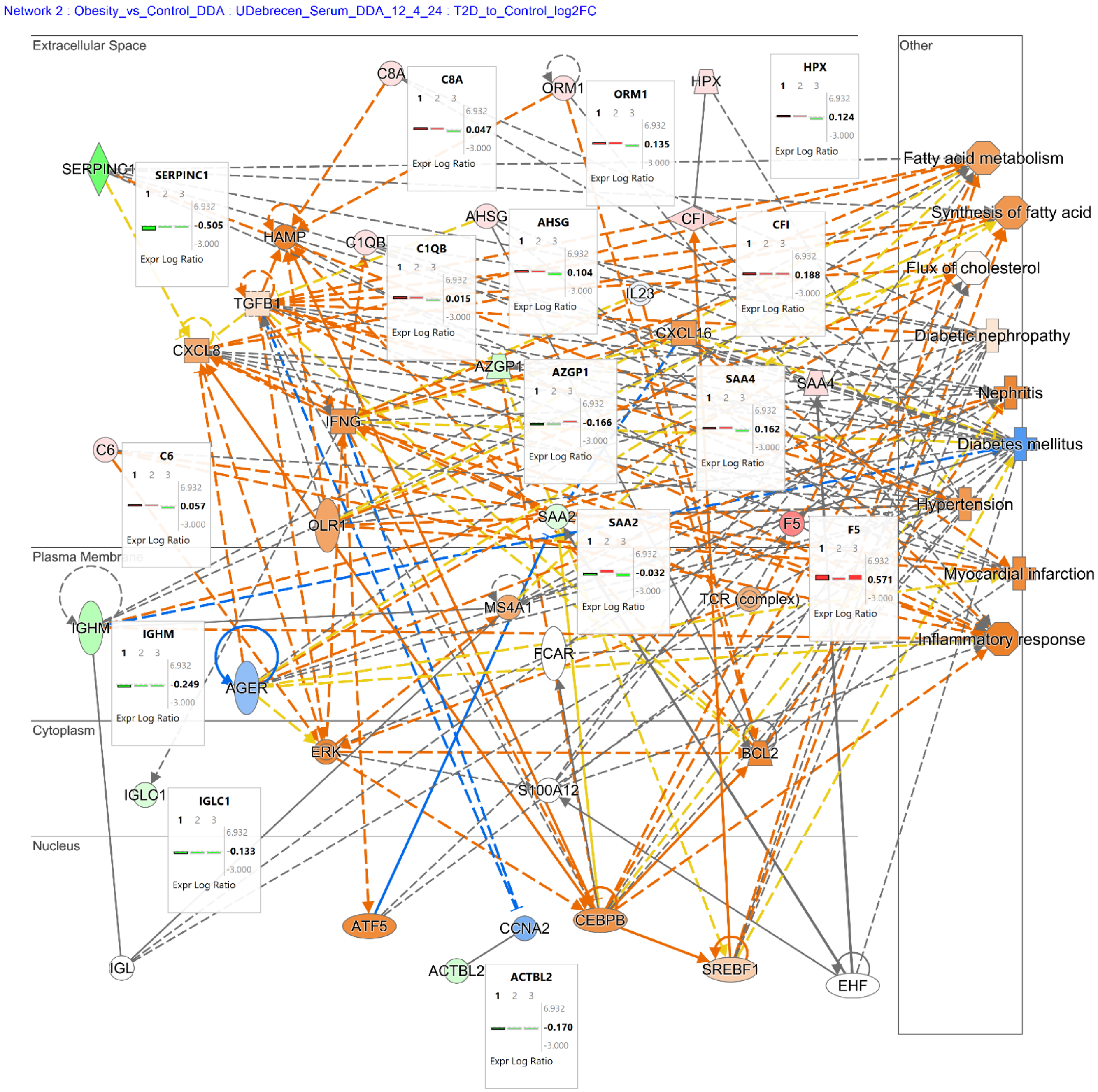

© 2000-2025 QIAGEN. All rights reserved.

T2D versus Obesity (Network 1). Network 1 : Obesity_vs_Control_DDA : UDebrecen_Serum_DDA_12_4_24 : T2D_to_Obesity_log2FC

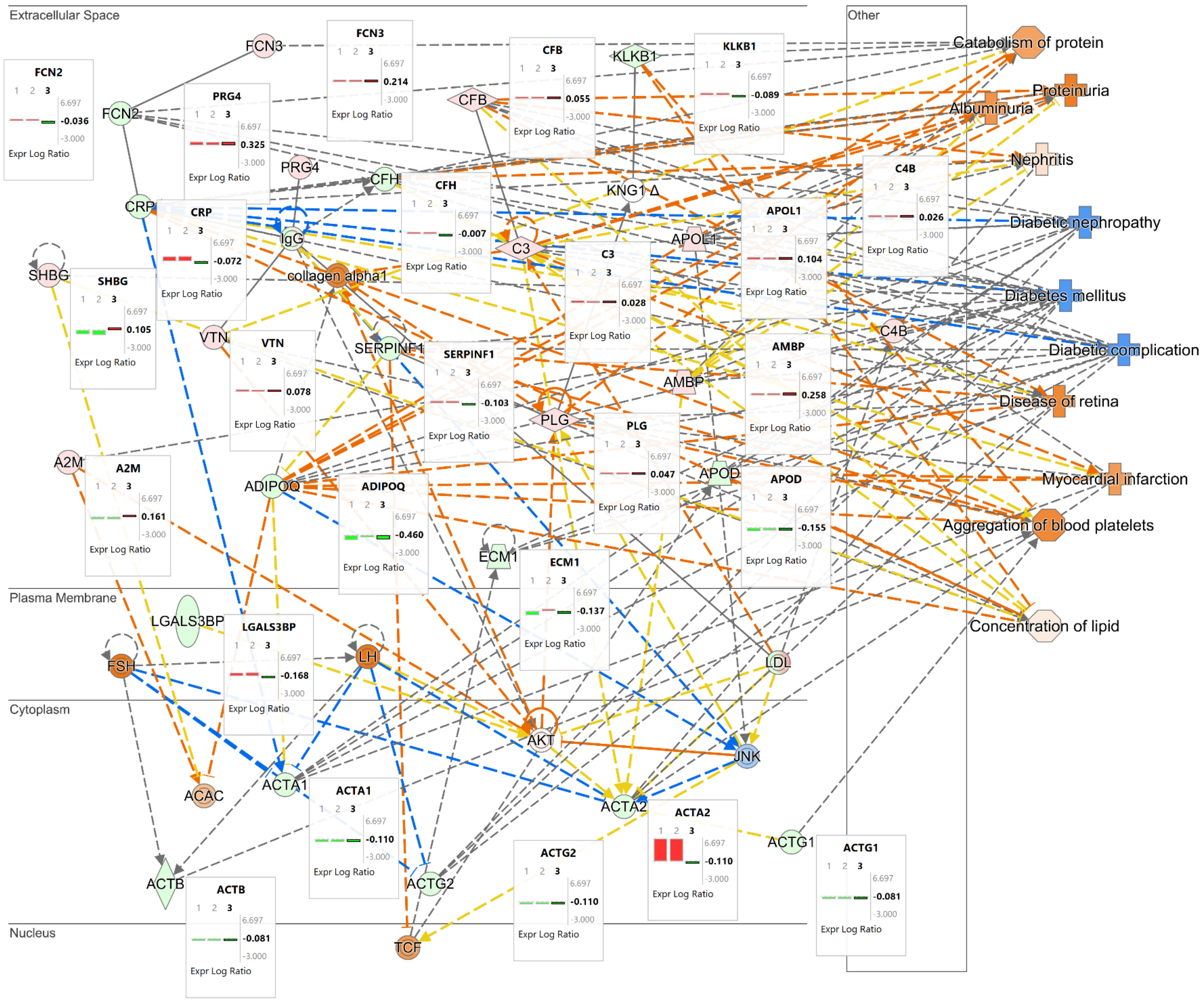

© 2000-2025 QIAGEN. All rights reserved.

T2D versus Obesity (Network 2).

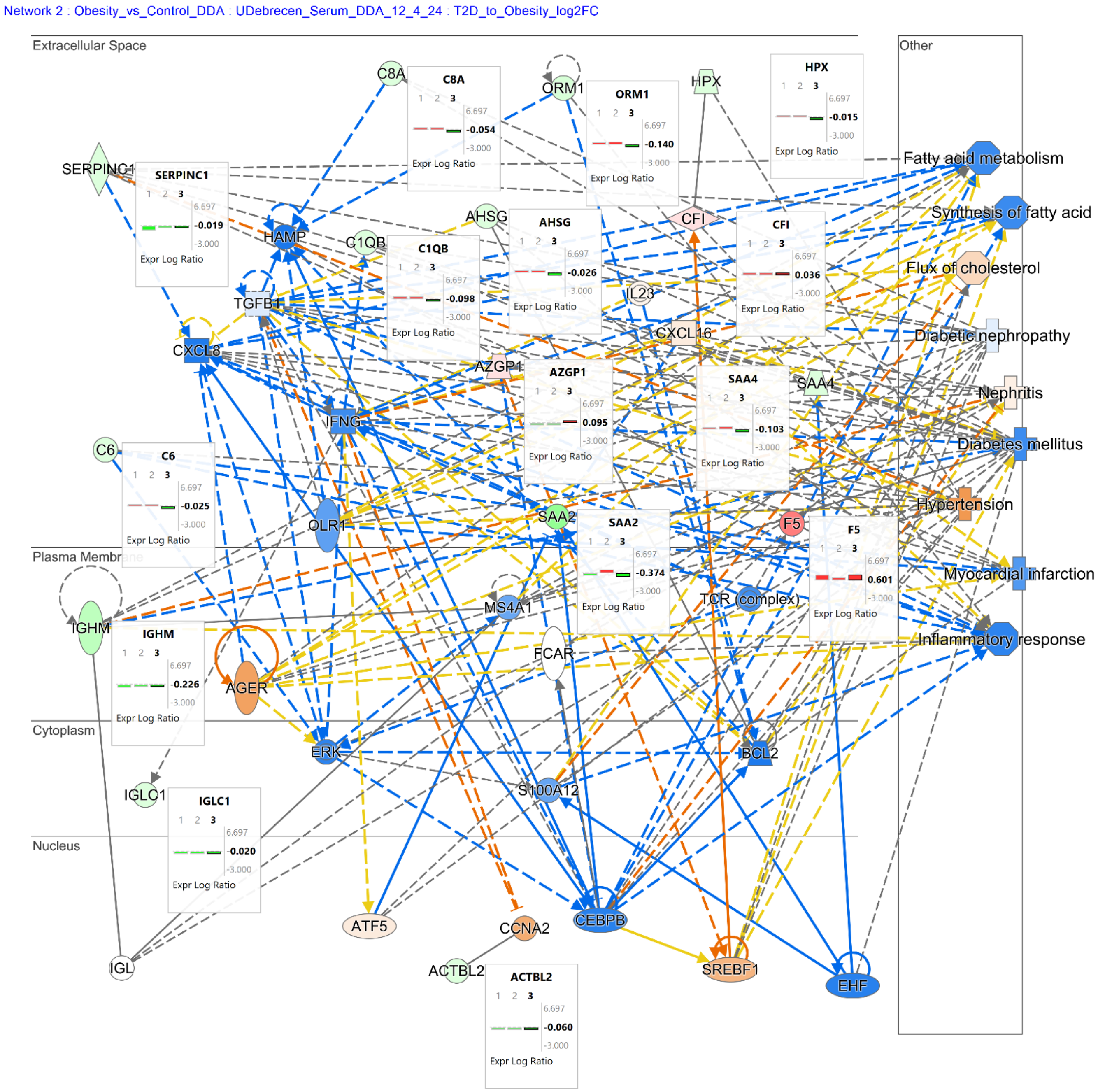

© 2000-2025 QIAGEN. All rights reserved.
