# Supplementary material for: Comprehensive Insights into Obesity and Type 2 Diabetes from Protein Network, Canonical Pathway, Phosphorylation and Antimicrobial Peptide Signatures of Human Serum
Source: Proteomes. 2025 Dec 17;13(4):67. doi: 10.3390/proteomes13040067 (PMC12736859; doi:10.3390/proteomes13040067)
Supplement: Supplementary file 1 [file proteomes-13-00067-s001.zip › Supplementary_Figure_S3.pdf]

Supplementary Figure S3. Protein-protein interaction networks of identified phosphoproteins generated by Ingenuity Pathway Analysis®.

Obesity versus Control (Network 1).

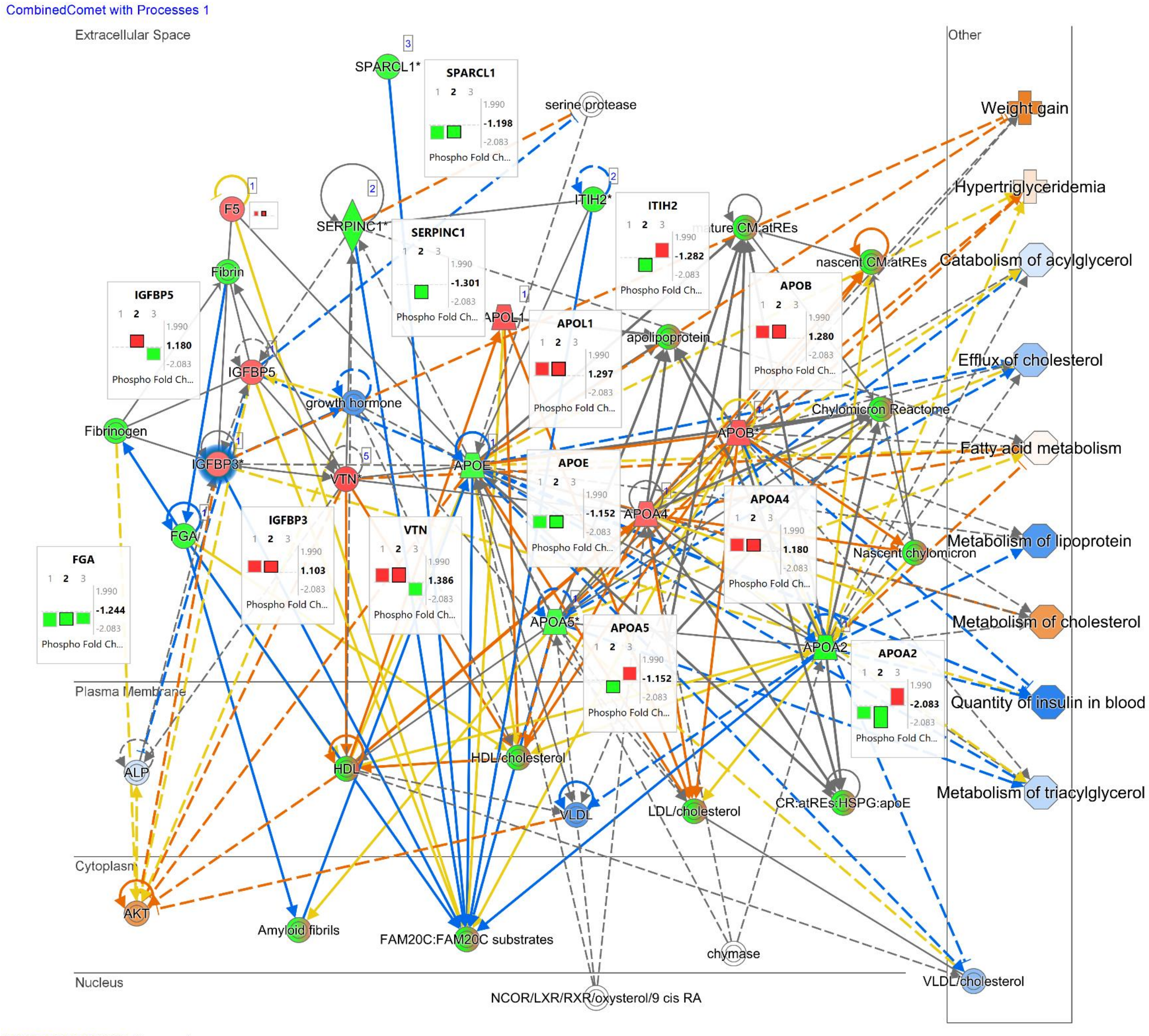

© 2000-2025 QIAGEN. All rights reserved.

Obesity versus Control (Network 2).

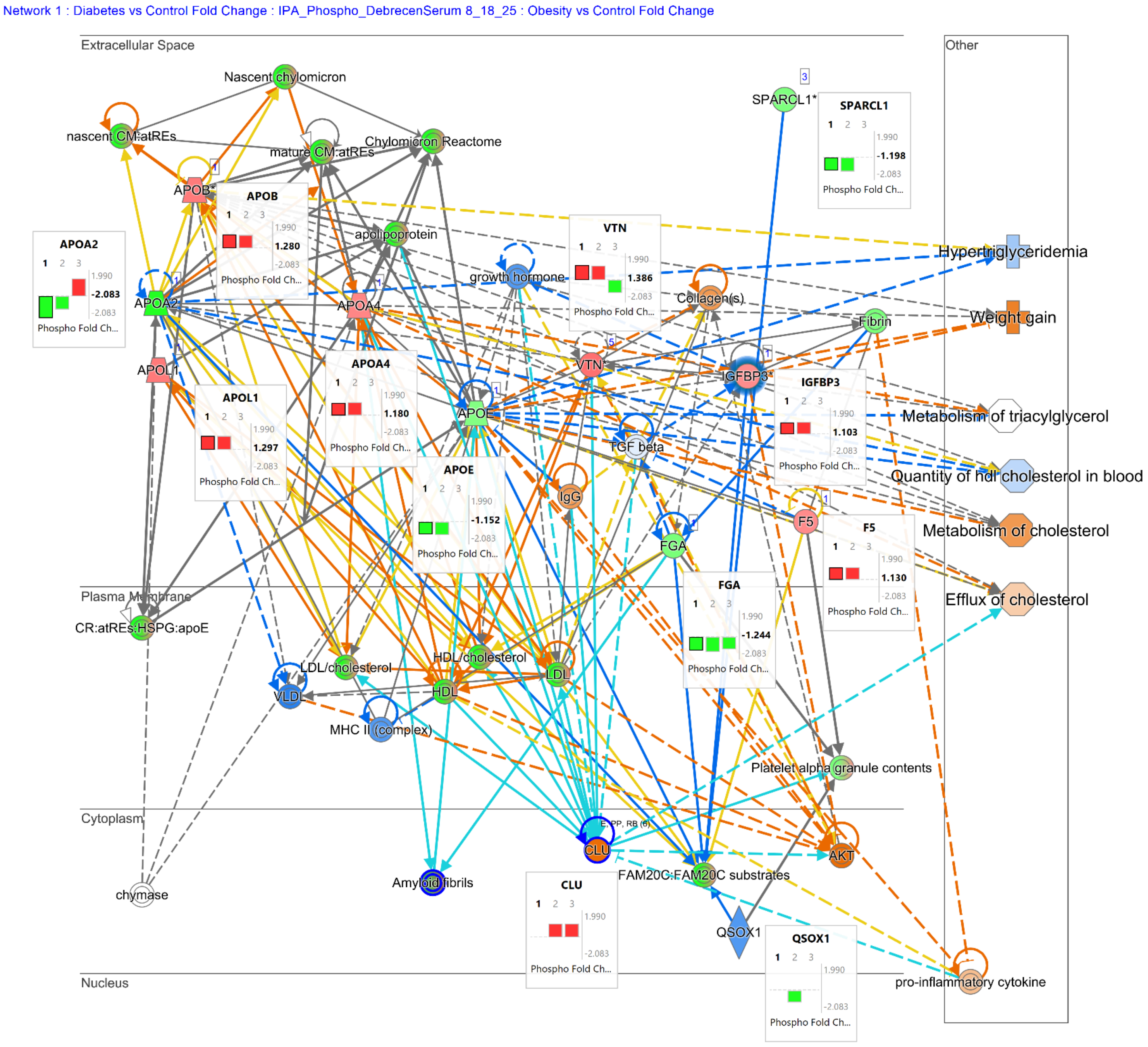

© 2000-2025 QIAGEN. All rights reserved.

T2D versus Control (Network 1).

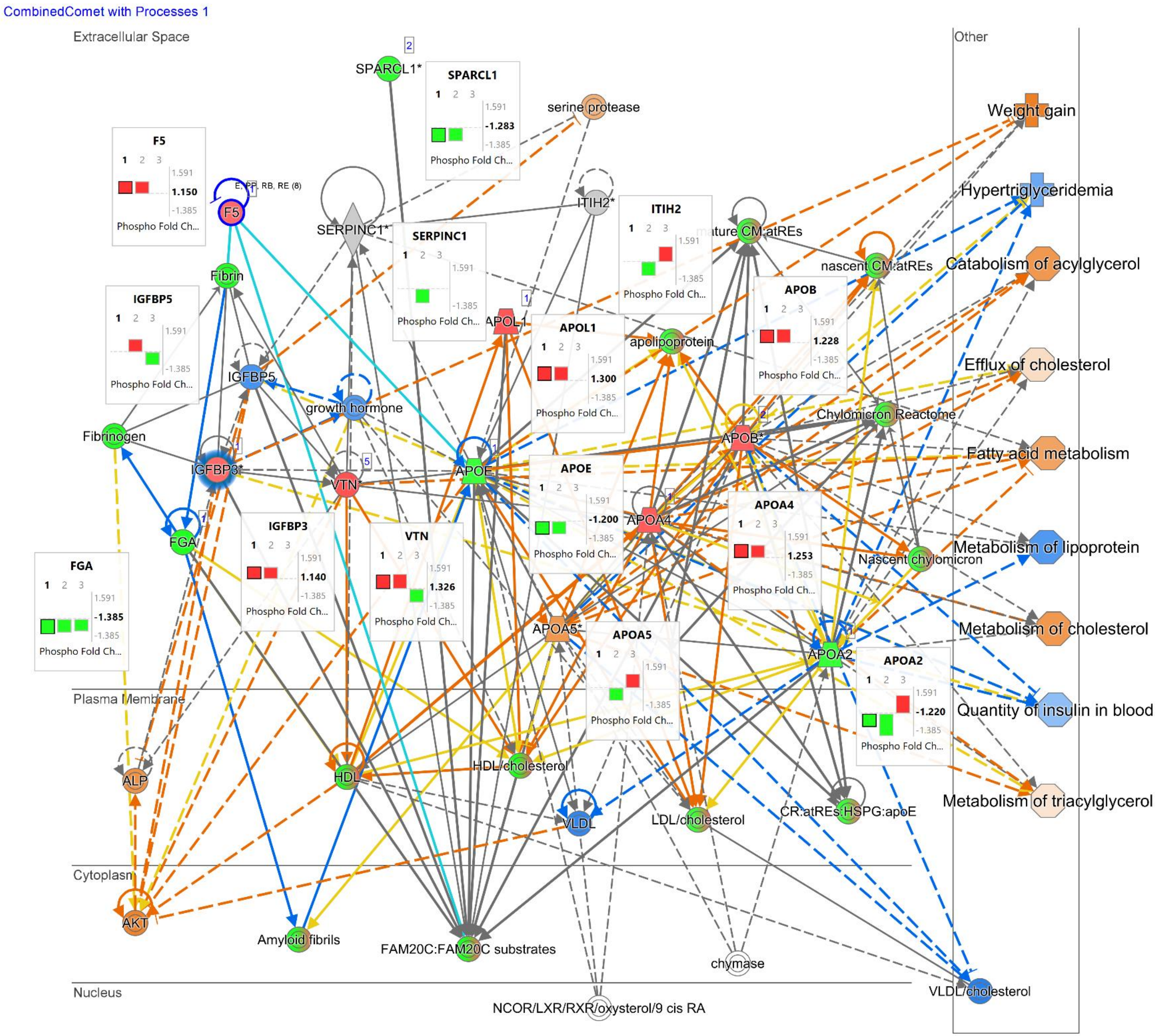

© 2000-2025 QIAGEN. All rights reserved.

T2D versus Control (Network 2).

Network 1 : Diabetes vs Control Fold Change : IPA_Phospho_DebrecenSerum 8_18_25 : Diabetes vs Control Fold Change

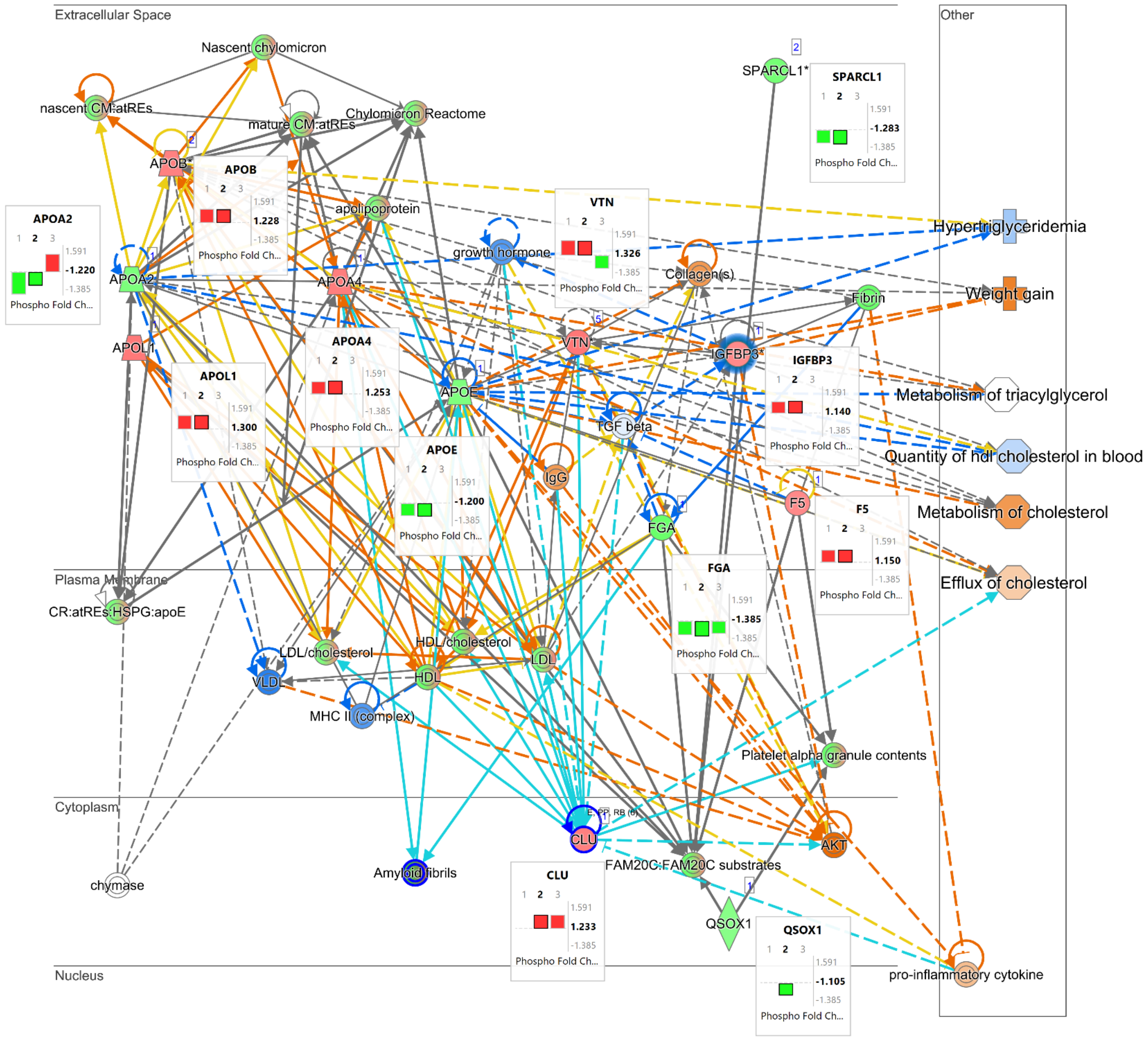

© 2000-2025 QIAGEN. All rights reserved.

T2D versus Obesity (Network 1).

CombinedComet with Processes 1

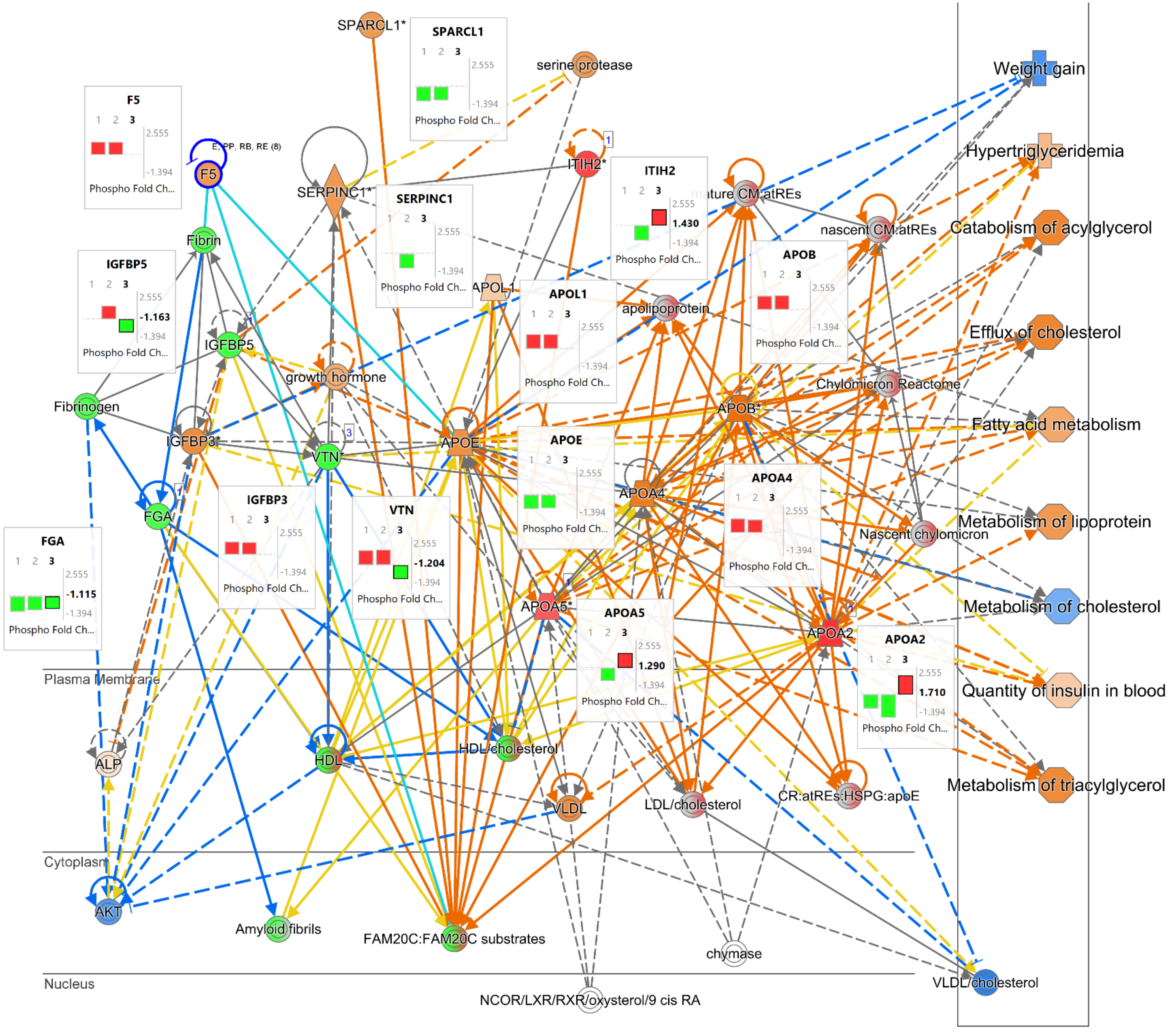

© 2000-2025 QIAGEN. All rights reserved.

T2D versus Obesity (Network 2).

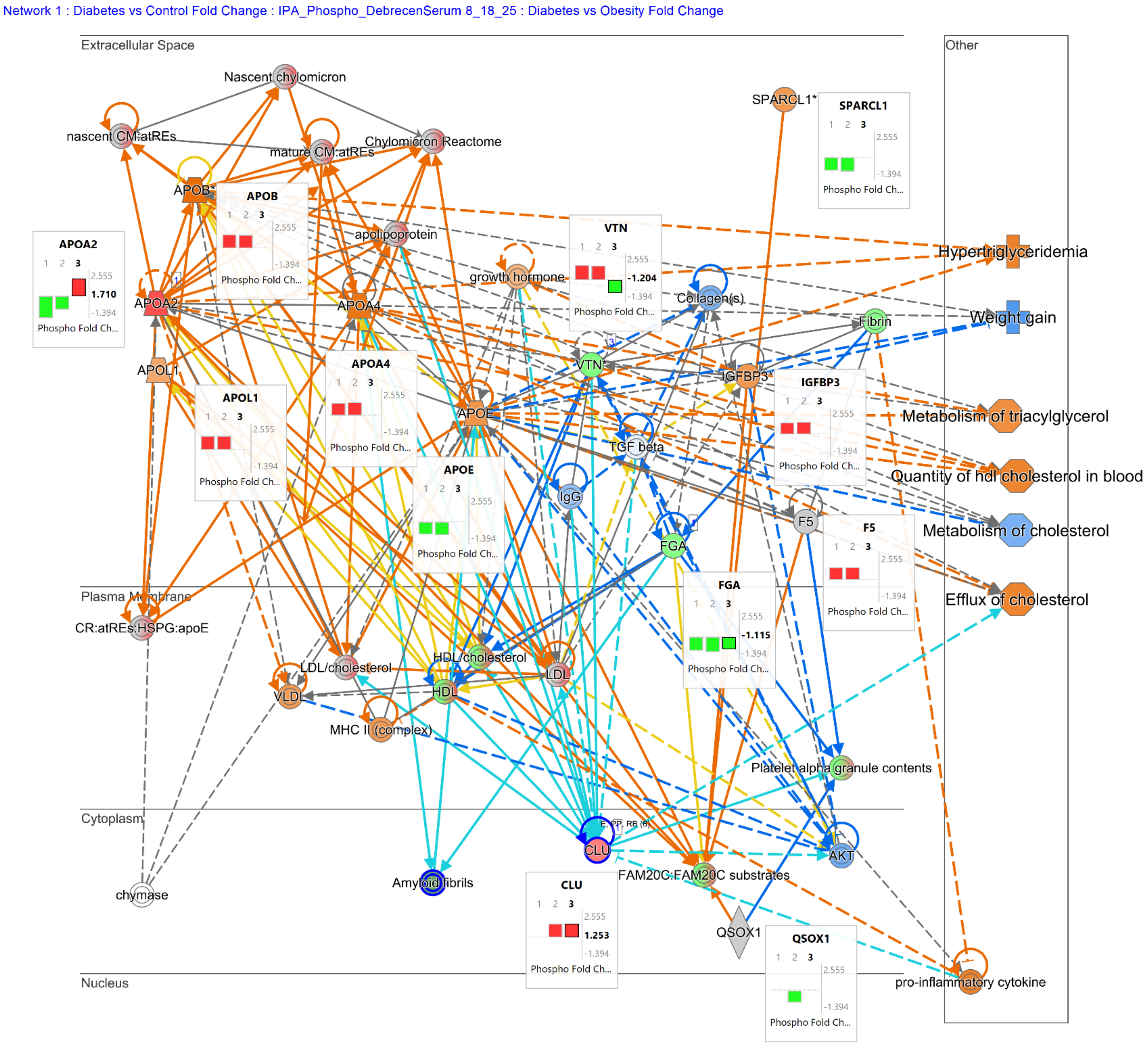

© 2000-2025 QIAGEN. All rights reserved.
