# Supplementary figures and images for: Comprehensive Insights into Obesity and Type 2 Diabetes from Protein Network, Canonical Pathway, Phosphorylation and Antimicrobial Peptide Signatures of Human Serum
Source: Proteomes. 2025 Dec 17;13(4):67. doi: 10.3390/proteomes13040067 (PMC12736859; doi:10.3390/proteomes13040067)

**Supplementary Figure S1.** Applied workflow for sample preparation and data analysis.

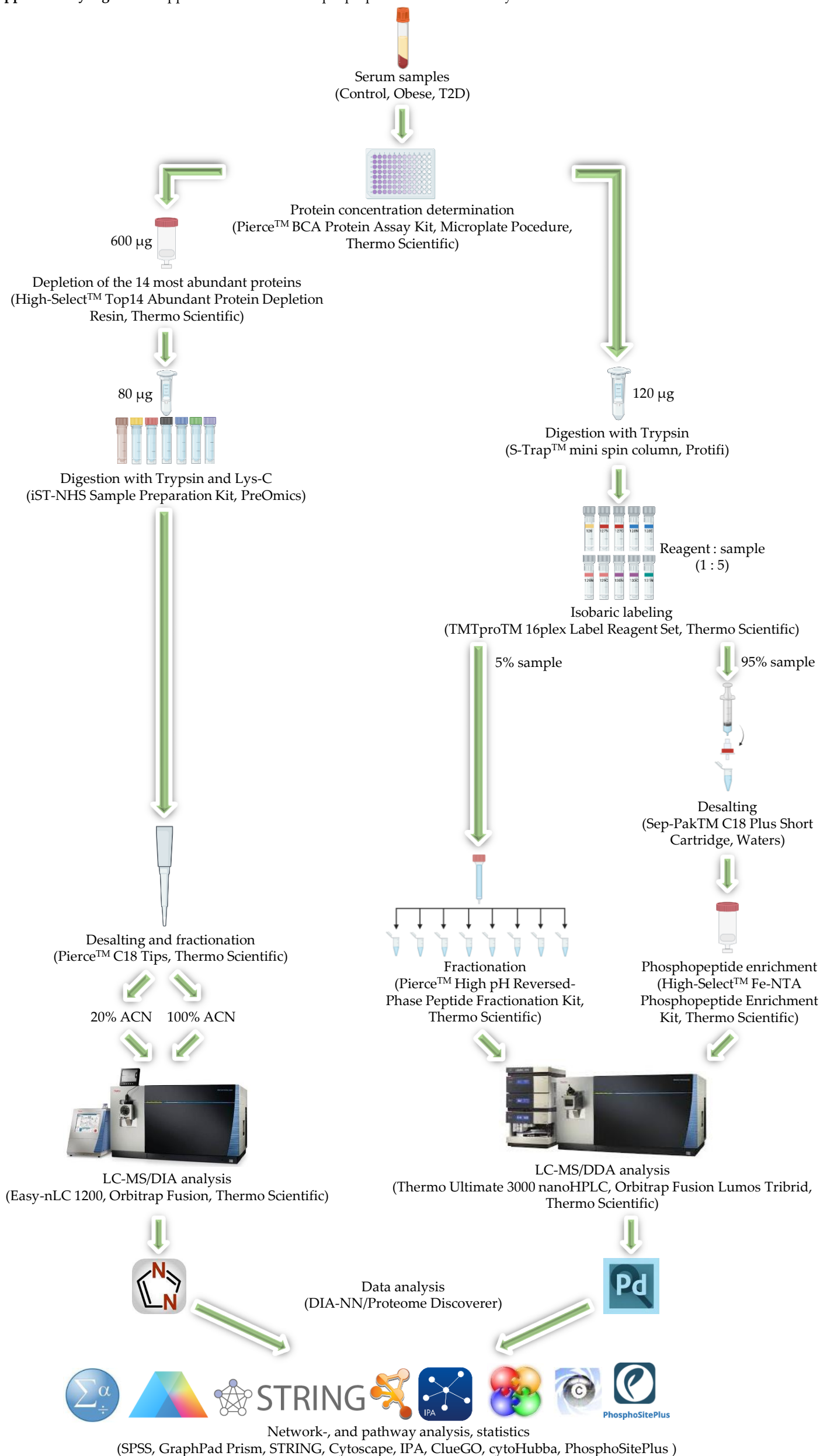

Supplement: Supplementary file 1 [file proteomes-13-00067-s001.zip › Supplementary_Figure_S1.pdf]
